# Influence of Selected Factors on the Relationship between the Dynamic Elastic Modulus and Compressive Strength of Concrete

**DOI:** 10.3390/ma11040477

**Published:** 2018-03-22

**Authors:** Krystian Jurowski, Stefania Grzeszczyk

**Affiliations:** Opole University of Technology, 45-758 Opole, Poland; s.grzeszczyk@po.opole.pl

**Keywords:** concrete, dynamic elastic modulus, elastic modulus, non-destructive testing

## Abstract

In this paper, the relationship between the static and dynamic elastic modulus of concrete and the relationship between the static elastic modulus and compressive strength of concrete have been formulated. These relationships are based on investigations of different types of concrete and take into account the type and amount of aggregate and binder used. The dynamic elastic modulus of concrete was tested using impulse excitation of vibration and the modal analysis method. This method could be used as a non-destructive way of estimating the compressive strength of concrete.

## 1. Introduction

The compressive strength and modulus of elasticity are the most important properties of concrete from the viewpoint of structural design. Commonly, these parameters are determined by the uniaxial compression of cylindrical or cube-shaped specimens, according to standard procedures, and so values obtained that way are considered to be the reference values. However, it is not always possible to use these methods in practice because they are destructive and require the collection of numerous test samples during concreting operations. In addition, properly conducted modulus of elasticity testing using cylindrical specimens is a relatively time-consuming process. Therefore, we seek non-destructive methods in order to estimate these parameters for hardened concrete, such as: the sclerometric method, the ultra-sound pulse velocity method, and the impulse excitation and modal analysis method.

Technological developments and better access to more appropriate apparatus has resulted in significant progress in the dynamic testing methods used in structural health monitoring. These methods could also be used in concrete material parameter testing (especially compressive strength) or to monitor its increase over time. A significant disadvantage of non-destructive methods is the fact that the values are achieved indirectly, i.e., the results of each test have to be converted to a certain parameter (e.g., the compressive strength of concrete) using a previously assumed relationship between these values. 

The natural frequencies obtained using impulse excitation testing and modal analysis methods are very good indicators of the state of concrete elements because the fundamental resonant frequency of vibration decreases with an increasing degree of material degradation [1]. This also allows monitoring of the concrete parameters as they change over time, especially in the first few days after sampling [2]. As mentioned above, results obtained through non-destructive methods have to be converted to the parameters required, using experimentally obtained relationships. The relationship between the compressive strength and modulus of elasticity of concrete has been the subject of numerous investigations, which have resulted in many equations linking these parameters. 

**Figure 1:**
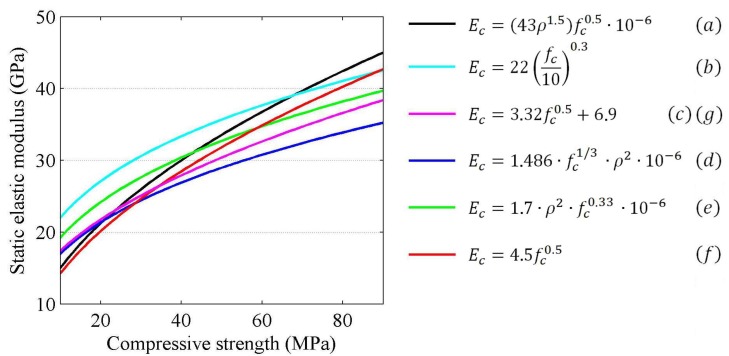
The selected relations between compressive strength and elastic modulus of concrete: (**a**) ACI 318-95; (**b**) Eurocode 2; (**c**) ACI 363R-92; (**d**) Noguchi et al. (2009); (**e**) BS 8110-2:1985; (**f**) CSA A23.3-04; (**g**) NZS 3101-2006 [4,5,6,7,8,9,10].

The most commonly used equations were presented by Neville (2000) [3]. Selected equations from the different standards are presented in Figure 1. These were derived by matching the coefficients of predetermined equations (commonly exponential or similar functions) in order to obtain the best fit model for the experimental results.

The equations mentioned above are convenient to use when calculating the compressive strength for a known modulus of elasticity because the compressive strength and concrete density are the only parameters required however, their accuracy in predicting the modulus of elasticity value is not sufficient for all kinds of concrete. This indicates that the relationship between elastic modulus and compressive strength is influenced by a number of factors, such as: the type of aggregate, humidity, age of the concrete, and the type of binder used. As preliminary research shows [11], the equations which take into account the value of static modulus of elasticity as well as concrete density provide better accuracy. However, the values calculated in this way could also be significantly different from the ones determined experimentally. It should be noted that concrete density is strongly correlated to the type of aggregate used.

The effect of aggregate type on the elastic properties of concrete is widely discussed in the literature e.g., [12,13]. These authors demonstrated the effect of aggregate type and also its volume content in concrete. Different models, treating concrete as a two phase material, were also considered [14]. While the effect of aggregate on the relationship between compressive strength and modulus of elasticity seems to be recognized, the influence of cement matrix type on this dependency is discussed in very few publications.

A comprehensive analysis, based on the results of more than 3000 concrete samples, is presented in paper [7]. Those authors proposed a relationship taking into account the aggregates used and the binder type. These parameters were determined for different types of aggregate and for selected kinds of binders. 

An interesting model is presented in [15]. The M5′ algorithm was used to achieve a multi-parameter relationship and this allows the Young’s modulus of concrete to be estimated. The M5′ is one of the algorithms in the model tree method which is relatively easy to use; this is further described in [16]. The proposed model is based on equations derived from many experimentally obtained results and application of the M5′ model tree algorithm indicates which one is appropriate for a specific kind of concrete. It should be mentioned that the paper refers to concrete containing recycled aggregate.

The values calculated using the equations proposed in the literature usually provide satisfactory results for specific kinds of concrete only. On the rare occasion that an attempt has been made to provide relationships that take into account the influence of binder and aggregate type, the results have been significantly different from the ones determined experimentally. Additionally, most of the equations proposed in the literature refer to ‘normal’ strength concrete, although high strength concrete is also investigated [17].

An additional problem when calculating the compressive strength of concrete using the impulse excitation method is the difference between the static and dynamic modulus of elasticity of concrete. The studies clearly show that the dynamic modulus is significantly greater than the static one. It could be the result of large differences in the strain rates associated with the static and dynamic testing methods [18] or it could be a result of the multi-phase nature of concrete [19].

**Figure 2:**
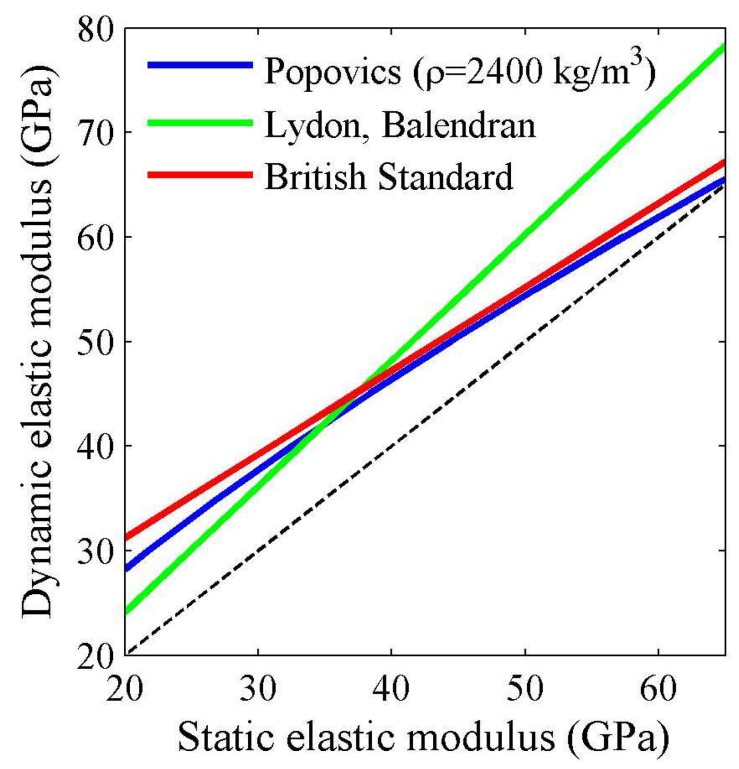
A relationship between the static and dynamic elastic modulus of concrete proposed by: Lyndon and Balendran [20], Popovics [21], and CP 110-1:1972 standard [22].

The literature survey revealed equations that describe the relationship between the static and dynamic modulus of elasticity. The most common are equations proposed by Lyndon and Balendran [20], Popovics [21], and by British Standard CP 110-1:1972 [22]. The relationship between the static and dynamic modulus of elasticity is presented in Figure 2. The estimation of the elastic modulus of concrete using non-destructive methods requires the application of a relationship between the dynamic and static elastic modulus as well as a relationship between the static elastic modulus and compressive strength of concrete.

The relationship between static and dynamic modulus was investigated e.g., in [23,24,25]. The authors demonstrate that this relationship depends highly on the specimen shape [23], concrete type and composition [24], as well as the testing methodology [25]. In [26], the relationship based on nonlinear deformation of concrete theory is proposed. The authors state that the relationship between static and dynamic modulus depends on the applied stress level in the static test as well as on the rate of loading.

The comprehensive study of the relationship between dynamic elastic modulus and compressive strength of concrete is presented in [27]. The authors demonstrate that the main factors which affect the relationship between static and dynamic elastic modulus are aggregate volume content and maximum size of the coarse aggregate. On the other hand, the water to cement ratio and curing temperature had no significant influence. The authors propose a linear relationship between static and dynamic elastic moduli.

The method of dynamic elastic modulus testing preferred in this paper is based on excitation of a concrete specimen using a hammer and then analysis of its dynamic response. Recommendations for concrete testing methods using impulse excitation methods and modal analysis can be found in [28] and the standards [29,30,31]. A comparison of these standards shows that all of them present similar testing procedures. The main differences are concerned with calculating correction factors to account for the finite thickness of a specimen.

**Figure 3:**
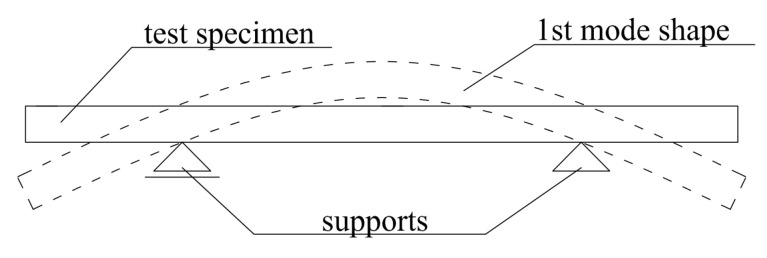
Free-free beam configuration.

It is most convenient to test beam-shaped samples of concrete. Commonly, these specimens are supported in vibration nodes to form a so-called ‘free-free’ configuration (Figure 3). In the case of testing concrete samples which are relatively blocky, it is acceptable to support them on rubber pads, in order to isolate the sample from the ground. The investigation into how this form of support influences the dynamic elastic modulus results was described in [32]. It was demonstrated that the resonant frequencies of concrete beams supported on rubber pads are about 2% higher when compared to beams supported on steel supports in vibration nodes. This difference is more significant in the case of relatively slender beams. In addition, it was stated that the location of the accelerometer (on the edge or in the middle of the span) had no significant effect on the value of fundamental vibration frequency of concrete samples.

Non-destructive methods of concrete testing are commonly based on experimentally derived relationships. The accuracy of the methods mentioned is highly dependent on the assumptions made. The literature survey, as well as experimental tests, demonstrated that widely used relationships are not appropriate to all types of concrete, especially in the case of concrete containing mineral additives.

In case of concrete at early-age, values of compressive strength and elastic modulus increase significantly. In practice, it is of interest to know when the framework can be removed or when a construction element can be loaded without excessive deflections or cracking. Therefore, non-destructive methods are highly demanded in the situ testing. The accuracy of the estimated values in this way depends on the relationship between dynamic elastic modulus (obtained from the non-destructive testing), static elastic modulus, and compressive strength. In this paper, the investigation of different concrete compositions and properties, including different types of cement matrix, is presented. Based on static and dynamic elastic modulus and compressive strength testing results, the relationship between these parameters was formulated, taking into account the age of the concrete and the type and volume content of each ingredient (especially binder type). The impulse excitation and modal analysis method were used as a non-destructive method to determine the dynamic elastic modulus of concrete. It should be noted that values of initial and stabilized elastic modulus (introduced recently in EN 12390-13:2014 standard) were included to the proposed relationship between elastic modulus and compressive strength of concrete, which is the novelty in this field.

## 2. Materials and Methods

In order to formulate the relationship between dynamic elastic modulus and compressive strength, different types of concrete were investigated. The tested concrete compositions varied in terms of the amount and type of each aggregate and also the type of binder. The chemical composition of the cements and metakaolin used is presented in Table 1.

In concrete mixes M1–M3, the same aggregate type (natural aggregate) and the same volume content of aggregate were maintained. These mixes varied in the type of binder used. In order to increase the effect of cement type on the elastic properties of the concrete, an increased binder content was applied. This was carried out on self-compacting concrete (M4 and M5) and high strength concrete (M6).

**Figure 4:**
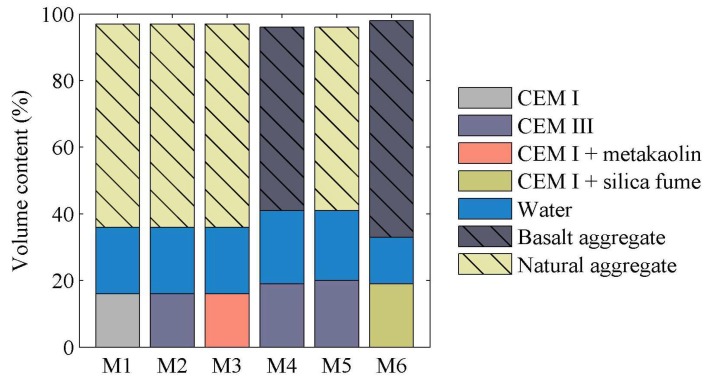
Volume composition of concrete mixes.

The concrete mixes M4 and M5 varied only by the aggregate type. The same volume content of basalt aggregate (M4) and natural aggregate (M5) was applied. The composition of concrete M1–M6 is presented in Table 2. Figure 4 presents the volume composition of the M1–M6 mixes. For all designed concrete mixes the compressive strength, static, and dynamic elastic modulus testing was conducted in a timescale of 4 to 28 days. 

The dynamic elastic modulus was determined using procedures based on EN ISO 12680-1:2008. In this method, the vibration of the concrete beam is induced by a random force hitting the beam. The recorded signal is subjected to fast Fourier transform (FFT) and the fundamental resonant frequency was then determined.

**Figure 5:**
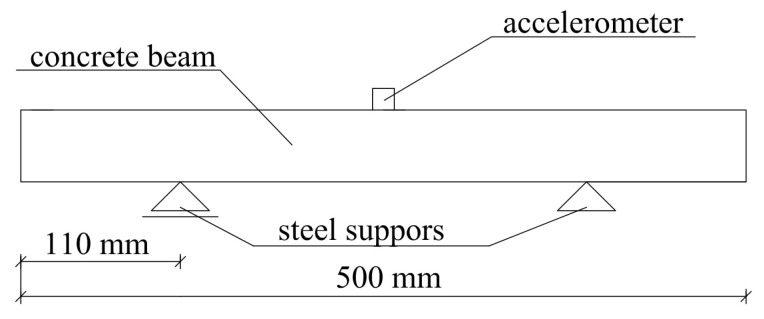
Scheme of test stand used for testing dynamic elastic modulus.

The dimensions of the concrete samples were: 500 mm length, 100 mm width, and 100 mm height. Each specimen was supported by steel tubes, positioned in such a way so as to coincide with the theoretical occurrence of fundamental vibration mode nodes. An illustration of the apparatus used in the dynamic elastic modulus testing is presented in Figure 5.

The values of dynamic elastic modulus were calculated using the analytical relationship between the rigidity of the test sample, its geometry and mass and the fundamental resonant frequency, using the equation

(1) $$E_{D}=\frac{\rho }{I}{\left(\frac{2\pi \cdot f\cdot L^{2}}{\beta }\right)}^{2}$$

where $E_{D}$ is the dynamic elastic modulus in Pa, f—is the fundamental resonant frequency in Hz, L—is the length of the tested specimen in m, I—is the cross section moment of inertia in m^4^, and β—is a constant that is dependent on the vibrating mode (equal to 22.373). In order to take into account such factors as the finite thickness of the specimen and Poisson’s ratio, the results of Equation (1) were multiplied by the correction factor, according to [26].

Compressive strength was determined using the method described in European standard EN 12390-3.

The static elastic modulus was determined using cylindrical specimens—150 mm diameter and 300 mm high—in accordance to EN 12390-13:2014. Standard method ‘A’ was applied and then two kinds of static elastic modulus were determined: initial and stabilized (designated as *E_C_*_,0_ and *E_C_*_,*S*_, respectively). The parallelism of the top and bottom surfaces was obtained using the steel caps and fine sand layer method, in accordance with Appendix ‘A’ of PN-EN 12390-3.

The specimens used in all of the experiments were cured in water at a temperature of 20 °C and then tested in the surface-dried state, from 4 to 28 days. 

## 3. Experimental Results and Discussion

**Figure 6:**
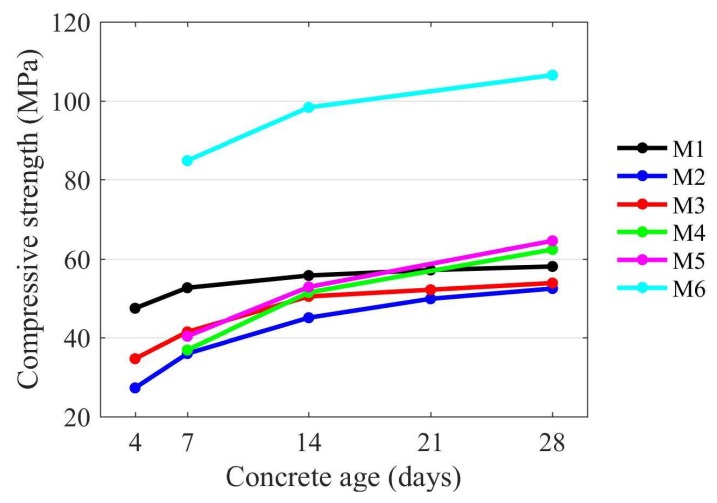
Compressive strength of concrete mixes M1–M6.

**Figure 7:**
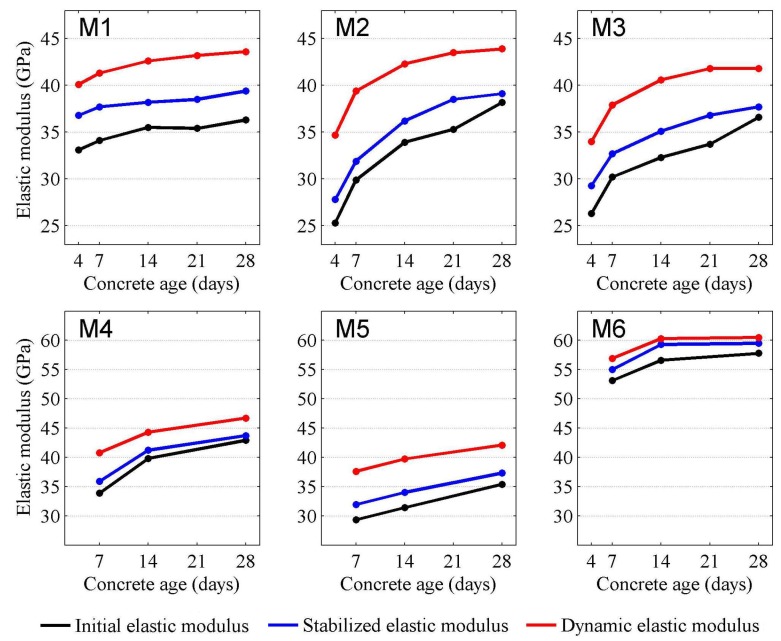
The comparison of static and dynamic elastic modulus of concrete mixes M1–M6.

The results of compressive strength and static and dynamic elastic modulus testing are presented in Table 3. The selected trends in the results achieved are presented in Figure 6 and Figure 7.

As Figure 6 shows, after 28 days of curing, the compressive strength exceeds 50 MPa for all of the concrete tested. The highest values of compressive strength were reached by the high strength concrete (M6). The self-compacting concrete (M4 and M5) achieved similar compressive strength (approximately 60 MPa) although they were made using a different type of aggregate.

The comparison of static and dynamic elastic modulus (Figure 7) shows that, for the time interval of 4 to 28 days, the values of initial static elastic modulus were lower and proportional to the stabilized elastic modulus for all of the concrete tested. In the case of M1–M3 concrete, the difference between these values is approximately 10%, regardless of age. Considering the M1–M3 concrete mix composition, indicates that the *E_C_*_,0_/*E_C_*_,*S*_ ratio depends on the aggregate type rather than cement matrix.

**Figure 8:**
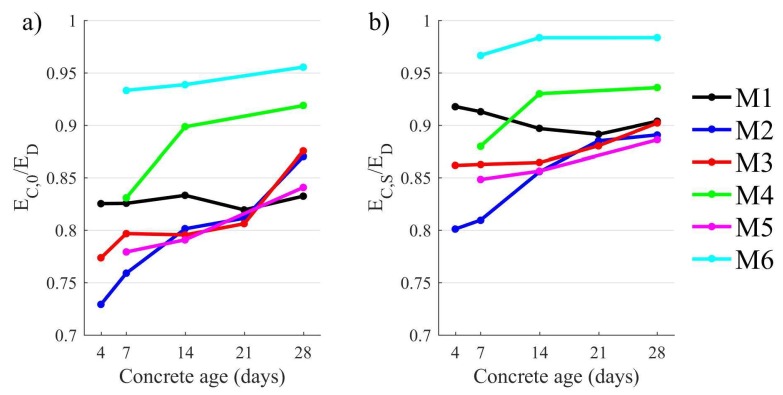
The relative differences between dynamic and static elastic modulus of concrete: (**a**) initial; (**b**) stabilized.

Figure 8 shows the differences between the initial and stabilized static elastic modulus and dynamic elastic modulus of concrete obtained using the impulse excitation and modal analysis method. It could be concluded that these differences generally decrease with time and this could be connected with increasing compressive strength. It should be stressed that relative differences between static and dynamic elastic modulus are smallest in the case of high strength concrete (M6), where these values tend to be nearly equal.

## 4. Relationship between Elastic Modulus and Compressive Strength

In order to formulate the relationship between the dynamic elastic modulus, obtained using the impulse excitation and modal analysis method, and compressive strength of concrete, we first need to establish the relationship between dynamic elastic modulus ($E_{D}$) and initial static modulus ($E_{C,0}$). In order to take into account the concrete’s composition, Equation (2) is proposed, in which α and $\beta_{cc}$ are functions related to the kind of aggregate and the type of binder, respectively.

(2) $$E_{C,0}=E_{D}\cdot \alpha \cdot \beta_{cc},$$

where

(3) $$\alpha =1.5\cdot \gamma \cdot k_{k},$$

where γ is the volume content of aggregate in the concrete mix, $k_{k}$ is the proportionality ratio between the initial ($E_{C,0}$) and stabilised static elastic modulus ($E_{C,S}$) depending on the kind of aggregate used. It was observed that these values appeared to be proportional (Figure 8), so it can be stated that

(4) $$E_{C,0}=E_{C,S}\cdot k_{k}.$$

The results show that the difference between $E_{C,0}$ and $E_{D}$ decreases with an increase in compressive strength. In order to take this into account, the function of the compressive strength increase ($\beta_{cc}$) was used. Originally, $\beta_{cc}$ is the function used to estimate the concrete’s compressive strength depending on its age, according to Eurocode 2 Equation (5). The parameters of this function are age (*t*) in days and the coefficient depends on the binder type ($s_{s}$)

(5) $$\beta_{cc}=\exp\left(s_{s}\left(1-{\left(\frac{28}{t}\right)}^{0.5}\right)\right).$$

**Figure 9:**
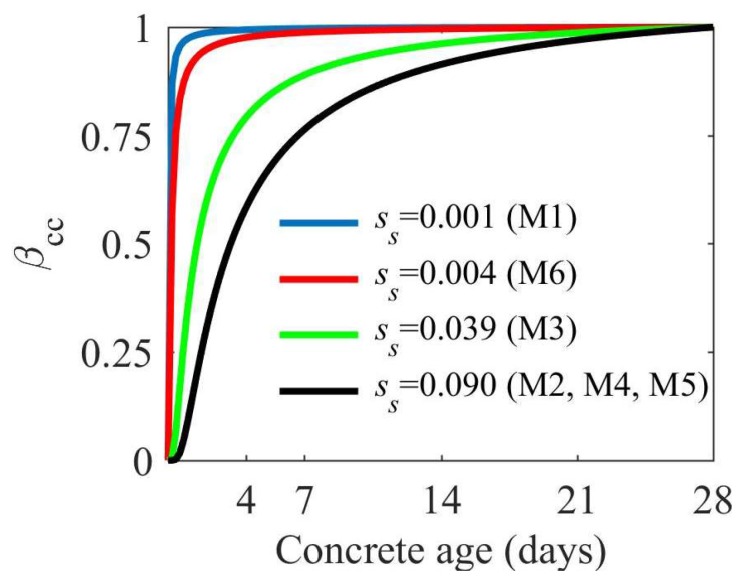
Increase in the compressive strength of tested concrete, as described by the $\beta_{cc}$ function.

This exponential function reflects the rate of increase in concrete parameters over time. The rate of this increase is regulated by the $s_{s}$ coefficient, which was determined for all binder types used in the tested concrete (M1–M6). Figure 9 presents the shape of the $\beta_{cc}$ function for the following binder types: CEM I, CEM III, CEM I with metakaolin, and CEM I with silica fume.

The values of the coefficients $s_{s}$ and $k_{k}$ were determined using the least-squares method, taking as a criterion the smallest difference between values of stabilized elastic modulus calculated using Equation (2) and those determined experimentally. The values of the $s_{s}$ and $k_{k}$ coefficients are presented in Table 4. 

Knowing the values of static elastic modulus ($E_{C,S}$) allows for the calculation of the compressive strength of concrete using the relationships outlined in the literature. In this work, the relationship given in ASTM 318-95 was used

(6) $$E_{C,S}=43\cdot \rho^{1.5}\cdot {f_{c}}^{0.5}\cdot 10^{-6},$$

where ρ is the density of concrete, thus the compressive strength is equal to

(7) $$f_{c}={\left(\frac{10^{6}\cdot E_{C,S}}{43\cdot \rho^{1.5}}\right)}^{2}.$$

Taking into account the coefficients that are dependent on the type of aggregate and binder (Table 4), the compressive strength was calculated using Equation (7). The calculated values were compared to the values obtained experimentally and these were then assessed using the Pearson correlation coefficient (R^2^ = 0.921). The largest differences between the calculated and experimental values are approximately 30%. This indicates that the relationship between static elastic modulus and compressive strength is influenced by the type of binder used. Thus, the parameter related to binder type and volume content was introduced to Equation (7)

(8) $$k_{s}(1-\gamma ),$$

where $k_{s}$—is the experimentally determined coefficient dependent on the type of binder and (1−γ) is the volume content of cement matrix in the concrete. The relationship then takes the form

(9) $$f_{c}=k_{s}(1-\gamma ){\left(\frac{10^{6}\cdot E_{C,S}}{43\cdot \rho^{1.5}}\right)}^{2}$$

The values of the $k_{s}$ coefficient for all of the binders used were calculated based on the investigation results (Table 3) and they are presented in Table 5.

**Figure 10:**
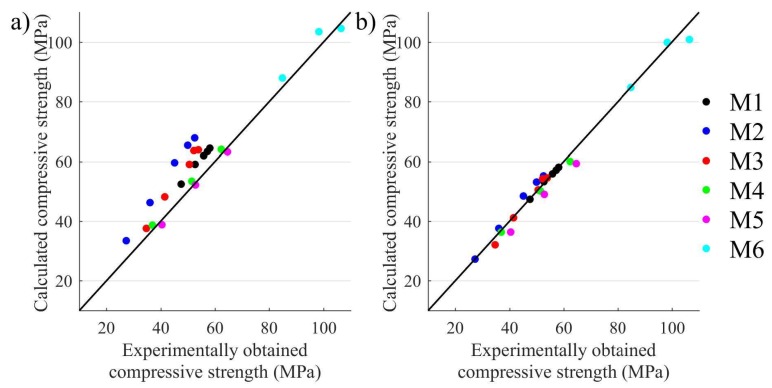
Comparison of calculated and experimentally obtained compressive strength of concrete values (**a**) using relationship (7); (**b**) using relationship (9).

The comparison of calculated compressive strength (according to ASTM 318-95 and using the relationship proposed in this paper) with the experimentally obtained values is presented in Figure 10. It is evident that introduction of the $k_{s}$ coefficient, which takes into consideration the type of binder Equation (9), allows a significantly improved correlation between the analyzed material parameters of concrete to be achieved. In this case, the Pearson correlation coefficient equals R^2^ = 0.950. 

Based on the investigations carried out, it can be stated that the proposed relationship allows the compressive strength of concrete to be calculated with better accuracy. The differences between calculated values and those obtained experimentally (Figure 10a) indicates the type of binder used and this influences the elastic properties of concrete significantly. 

## 5. Conclusions

Based on the results of our investigation, the relationship between the static and dynamic elastic modulus and the relationship between the static elastic modulus and compressive strength of concrete were formulated.

The proposed relationship between dynamic elastic modulus and compressive strength takes into account the type and amount of aggregate and binder type. It was demonstrated that consideration of these factors allows compressive strength to be calculated with better accuracy.

The investigation results showed that the stabilized elastic modulus is proportional to the initial elastic modulus and that the proportionality coefficient between them is dependent on the type of aggregate used.

The proposed relationship between dynamic elastic modulus and compressive strength could allow the use of impulse excitation and modal analysis method as a non-destructive method of compressive strength testing for concrete. However, the relationship should be developed through further experimental analysis of the results achieved for different types of concrete.

## Figures and Tables

**Table 1:** The chemical composition of cements and metakaolin.

Cement/Additive	*SiO*_2_	*Al*_2_ *O*_3_	*Fe*_2_ *O*_3_	*CaO*	*MgO*	*K*_2_ *O*	*Na*_2_ *O*	*SO*_3_	*TiO*_2_
	(%)								
CEM I	17.9	5.8	2.9	63.1	1.2	0.8	0.1	2.1	-
CEM III	28.8	6.9	1.8	51.0	5.3	0.6	0.3	1.9	-
Metakaolin	52.7	40.6	1.93	0.3	0.3	1.6	0.0	-	0.4

**Table 2:** Concrete mixes composition $\left(\mathrm{kg}/m^{3}\right)$.

Concrete	Binder				Water	Natural Aggregate	Basalt Aggregate	SP (%mass)
	CEM I	CEM III	Metakaolin	Silica Fume				
M1	500	-	-	-	200	1600	-	-
M2	-	501	-	-	200	1600	-	-
M3	387	-	97	-	193	1600	-	0.5
M4	-	582	-	-	232	-	1692	2.0
M5	-	585	-	-	202	1493	-	2.0
M6	500	-	-	50	135	-	2008	1.25

**Table 3:** The concrete specimen test results.

Concrete	Age (days)	Compressive Strength f_c,cube_	Static Elastic Modulus		Dynamic Elastic Modulus *E_D_*
			Initial $E_{C,0}$	Stabilized $E_{C,S}$	
		(MPa)	(GPa)	(GPa)	(GPa)
M1, CEM I, natural aggregate	4	47.5	33.1	36.8	40.1
	7	52.7	34.1	37.7	41.3
	14	55.8	35.5	38.2	42.6
	21	57.2	35.4	38.5	43.2
	28	58.1	36.3	39.4	43.6
M2, CEM III, natural aggregate	4	27.3	25.3	27.8	34.7
	7	36.0	29.9	31.9	39.4
	14	45.1	33.9	36.2	42.3
	21	49.9	35.3	38.5	43.5
	28	52.5	38.2	39.1	43.9
M3, CEM I + metakaolin, natural aggregate	4	34.7	26.3	29.3	34.0
	7	41.5	30.2	32.7	37.9
	14	50.5	32.3	35.1	40.6
	21	52.2	33.7	36.8	41.8
	28	53.9	36.6	37.7	41.8
M4, CEM III, basalt aggregate	7	36.9	33.9	35.9	40.8
	14	51.5	39.8	41.2	44.3
	28	62.4	42.9	43.7	46.7
M5, CEM III, natural aggregate	7	40.4	29.3	31.9	37.6
	14	52.9	31.4	34.0	39.7
	28	64.6	35.4	37.3	42.1
M6, CEM I + silica fume, basalt aggregate	7	84.8	53.1	55.0	56.9
	14	98.3	56.6	59.3	60.3
	28	106.5	57.8	59.5	60.5

**Table 4:** Determined values of the coefficients depending on aggregate and binder type.

Concrete	Aggregate	Binder	Concrete Density	Volume Content of Aggregate	Coefficients Determined Using Least Squares Method	
			$\rho \;\left(kg/m^{3}\right)$	γ	$k_{k}$	$s_{s}$
M1	Natural	CEM I	2360	0.61	0.926	0.001
M2	Natural	CEM III A	2340	0.61	0.926	0.090
M3	Natural	CEM I + metakaolin	2310	0.61	0.926	0.039
M4	Basalt	CEM III A	2560	0.55	0.964	0.090
M5	Natural	CEM III A	2320	0.55	0.926	0.090
M6	Basalt	CEM I + silica fume	2650	0.65	0.964	0.004

**Table 5:** The values of coefficient $k_{s}$

Binder	$k_{s}$
CEM I 42.5R	2.31
CEM III A 42.5N	2.09
CEM I + metakaolin	2.19
CEM I + silica fume	2.76
